# Polyostotic Fibrous Dysplasia With and Without McCune–Albright Syndrome—Clinical Features in a Nordic Pediatric Cohort

**DOI:** 10.3389/fendo.2018.00096

**Published:** 2018-03-15

**Authors:** Pauliina Utriainen, Helena Valta, Sigridur Björnsdottir, Outi Mäkitie, Eva Horemuzova

**Affiliations:** ^1^Children’s Hospital, Helsinki University Central Hospital, Helsinki, Finland; ^2^Children’s Hospital, University of Helsinki, Helsinki, Finland; ^3^Folkhälsan Research Center, Helsinki, Finland; ^4^Department of Women’s and Children’s Health, Karolinska Institute, Stockholm, Sweden; ^5^Department of Molecular Medicine and Surgery, Karolinska Institute, Stockholm, Sweden; ^6^Department of Clinical Genetics, Karolinska University Hospital, Stockholm, Sweden; ^7^Pediatric Endocrinology Unit, Astrid Lindgren’s Children Hospital, Karolinska University Hospital, Stockholm, Sweden

**Keywords:** fibrous dysplasia, polyostotic, McCune–Albright syndrome, mosaicism, bone fractures, craniofacial abnormalities, precocious puberty, café-au-lait spots

## Abstract

**Objective:**

Fibrous dysplasia (FD) presents as skeletal lesions in which normal bone is replaced by abnormal fibrous tissue due to mosaic *GNAS* mutation. McCune–Albright syndrome (MAS) refers to FD combined with skin (café-au-lait) and endocrine manifestations. This study describes the clinical childhood manifestations of polyostotic FD and MAS in a Nordic cohort.

**Patients and design:**

We retrospectively reviewed a cohort of pediatric patients (*n* = 16) with polyostotic FD with or without MAS diagnosed and followed in two Nordic Pediatric tertiary clinics between 1996 and 2017.

**Results:**

Half of the 16 patients with polyostotic FD presented with MAS. All patients with MAS (*n* = 8) had café-au-lait spots, and either gonadotropin-independent precocious puberty (PP) (girls; *n* = 5) or abnormal testicle structure (boys, *n* = 3). None manifested hyperthyroidism or growth hormone excess. Mild hypophosphatemia was common (11/16), but none had signs of hypophosphatemic rickets. Craniofacial bone involvement was found in 12 patients (75%); in 5 of these, skeletal lesions were limited to craniofacial area. One child with craniofacial disease had lost vision due to optic nerve damage. Eleven (69%) patients had sustained a fracture at FD lesion, over half of them requiring surgical fixation of the fracture, most commonly in the proximal femur. The first symptoms leading to FD/MAS diagnosis included skull/facial asymmetry (*n* = 4), PP (*n* = 3), abnormal gait (*n* = 3), pathologic fracture (*n* = 3), wide-spread café-au-lait spots (*n* = 1), headache (*n* = 1), and vision loss (*n* = 1).

**Conclusion:**

Polyostotic FD and MAS remain diagnostic and therapeutic challenges because of the broad clinical spectrum. Recurrent fractures, pain, and even vision loss may impair the quality of life in children with FD.

## Introduction

Fibrous dysplasia (FD) is a genetic, non-inheritable skeletal disorder in which normal bone is replaced by abnormal bone structure in the dysplastic FD lesions (1). FD is caused by somatic activating mutation in the *GNAS* gene, coding for the α-subunit of the stimulatory G protein (G_s_) (2, 3). These assumingly post-zygotic missense mutations cause genetic mosaicism and lead to constitutional activation of cAMP regulating G_sα_ in the affected cells (1, 3). G_sα_ is widely expressed, and therefore the disease manifestations are strikingly heterogenous.

The clinical spectrum of these conditions is broad, depending on the distribution of genetic mosaicism. Skeletal lesions with abnormal bone structure may present only in one bone (monostotic FD; in 70–85% of patients) or in multiple sites (polyostotic FD; 15–30%) and can lead to deformities, fractures, functional impairment, and pain (1). In patients with craniofacial FD, facial asymmetry is common, and compression of the optic nerve can cause impairment of vision. Skeletal lesions usually develop during the first decade of life (4). FD can occur alone or in combination with typical café-au-lait skin spots and/or endocrinopathies due to overproduction of hormones as a part of McCune–Albright syndrome (MAS) (5, 6). While café-au-lait spots are typically the earliest extra-skeletal manifestation in MAS, the most common endocrine manifestations include precocious puberty (PP), hyperthyroidism, and renal phosphate wasting with or without rickets/osteomalacia (7, 8). Acromegaly caused by growth hormone (GH) excess is also rather common manifestation in MAS (9). According to the clinical definition by Collins and coworkers, the phenotype of MAS can include any combination of skeletal, skin, and endocrine features (1). However, the original and stricter definition of MAS, which is still sometimes used (10, 11), requires the presence of all three manifestations—skeletal, skin, and endocrine—for diagnosis.

Polyostotic FD, and particularly MAS is a rare condition. The prevalence of MAS ranges between 1/100,000 and 1/1,000,000 with no ethnic differences in the disease frequency (6). The exact prevalence of FD is not known. MAS represents in approximately 5% of all FD patients, usually associating with polyostotic but occasionally also with monostotic FD.

Although each separate manifestation of MAS is well known, there are only few studies reporting the typical clinical presentation and outcome in pediatric patients with polyostotic FD. In this study, we describe clinical features of polyostotic FD and MAS in a Nordic cohort including all pediatric patients with polyostotic FD in two tertiary referral hospitals in Finland and Sweden.

## Subjects and Methods

### Subjects

Our retrospective cohort study included all pediatric and adolescent patients with polyostotic FD and MAS who were followed in two tertiary hospitals, both largest hospitals in the respective countries: Children’s Hospital, Helsinki University Hospital, Helsinki, Finland (*n* = 7) and Astrid Lindgren’s Children Hospital, Karolinska University Hospital, Stockholm, Sweden (*n* = 9). Patients had been referred to hospital care based on clinical reasons. For this study, we reviewed retrospectively the clinical data for all patients who were diagnosed and followed with polyostotic FD between 1996 and 2017 in Stockholm and between 2004 and 2017 in Helsinki. Due to centralized care, the cohort includes the majority of pediatric patients with polyostotic FD in these two countries. In Finland, Children’s hospital has been a referral center for the whole country for pediatric patients with rare bone diseases since 2004. In Sweden, most pediatric patients with polyostotic FD and MAS are diagnosed or followed at Karolinska University Hospital. We therefore estimate that our cohort includes close to 100% of the Finnish and over half of the Swedish pediatric patients with polyostotic FD and MAS during the study period. Diagnosis of polyostotic FD was based on clinical, radiological, and histological findings; no genetic analyses were performed upon diagnosis. The diagnosis of MAS was defined as polyostotic FD together with any extra-skeletal manifestation (café-au-lait spots and/or any endocrine manifestation).

### Methods

The clinical data were obtained from the hospital records. For growth evaluations, recently published Finnish growth curves (12) were used for both Finnish and Swedish patients because the growth patterns do not significantly differ between these two Nordic populations. A representative growth curve was available for 13/16 patients. This retrospective registry study was approved by institutional review boards of Helsinki University Hospital, Helsinki, and Karolinska University Hospital, Stockholm, and it was carried out in accordance with the Declaration of Helsinki. Individual patient data in the tables are only shown categorized (age; areas of affected bones; etc.) so that patients cannot be identified.

The descriptive statistics were generated using IBM SPSS Statistics software for Windows, Version 23.0 (IBM Corp., Armonk, NY, USA).

## Results

### Cohort Characteristics

Our cohort included eight pediatric patients with MAS and eight patients with polyostotic FD only. Based on the background population of 15 million, and approximately 20% (3 million) of them in pediatric age group, this corresponds an estimated prevalence of 1 per 375,000 for MAS. The study cohort included eight (50%) females and eight males. Median age at last follow-up visit was 13.5 years (range 5.3–18.9). Median follow-up time from the diagnosis was 8.6 years (range 0–15.9). The clinical characteristics are presented in Table 1.

**Table 1:** Clinical characteristics of the Nordic cohort of pediatric patients with polyostotic fibrous dysplasia (*n* = 16).

Patient	Age category (years) at first symptom	Time from first symptom (years) to dg	Presenting manifestation	MAS	Café-au-lait	Endocrine involvement	Hypophosphatemia^a^
1	6–9	0.0	Pathological fracture (femur)	−	−	−	+
2	6–9	0.0	Prominence of skull	−	−	−	+
3	3–6	0.0	Pain; abnormal gait	−	−	−	+
4	0–3	1.0	PP	+	+	+ PP	+
5	6–9	0.2	Impaired vision	−	−	−	+
6	0–3	0.4	Several café-au-lait skin patches	+	+	+ PP	+
7	6–9	0.6	PP	+	+	+ PP	+
8	0–3	0.4	Pathological fracture (femur)	+	+	Abnormal testicle consistence	+
9	6–9	2.8	Abnormal position of left eye (distally)	−	−	−	−
10	0–3	0.4	Pain; abnormal gait	+	+	Abnormal testicle consistence	+
11	0–3	7.8	PP	+	+	+ PP	+
12	0–3	0.4	Head asymmetry	−	−	−	−
13	0–3	10.8	Face asymmetry	−	−	−	−
14	3–6	0.0	Pathological fracture (tibia)	+	+	+ PP	−
15	3–6	0.1	Abnormal gait after trauma	+	+	Abnormal testicle consistence	+
16	6–9	3.0	Headache	−	−	−	−

*Age categories: 0–3 = from newborn to 2.9 years, 3–6 = from 3.0 to 5.9 years, and 6–9 = from 6.0 to 9.0 years*.

*MAS, McCune–Albright syndrome; PP, precocious puberty*.

*^a^Ever presented with hypophosphatemia compared with reference range for age*.

### Diagnostic Features and General Characteristics

The median age at diagnosis of polyostotic FD was 5.0 years (range 0.74–11 years), whereas the median age at first symptom of the disease was 3.5 years (range 0–8.5). Among the MAS patients, the median age at first symptom was 2.7 (0.25–7.3) and that at diagnosis 3.6 (range 1.8–8.1) years. In the patients with polyostotic FD only, median age at first symptom and at diagnosis were 6.0 (0.0–8.5) and 7.3 (0.7–11) years, respectively.

The first manifestation of the disease in polyostotic FD without MAS (*n* = 8) was facial or skull asymmetry (*n* = 4), abnormal gait (*n* = 1), pathological fracture (*n* = 1), headache (*n* = 1), and impaired vision (*n* = 1). In patients with MAS (*n* = 8), the first manifestation was PP in three girls, café-au-lait lesions in two girls, abnormal gait in two and fracture in one. In most children, the diagnosis of polyostotic FD or MAS was set within a few months of symptom onset. In one female subject, MAS first presented with vaginal bleeding in infancy, but diagnosis of MAS was made only several years later, at 8.1 years of age, when she manifested with skeletal symptoms. In addition, one girl presented with facial asymmetry already at birth but was diagnosed with craniofacial FD only at 10.8 years, and another girl with multiple café-au-lait spots in infancy was diagnosed with MAS at the age of 2 years, when she developed precocious gonadotropin-independent puberty.

### Skeletal Lesions, Fractures, and Orthopedic Surgery

To evaluate the affected skeletal areas, bone scintigraphy was performed in altogether nine subjects, in seven of seven Finnish and in two of nine Swedish patients. In most of the Swedish patients, plain radiographs were used to evaluate the distribution of FD lesions. In addition, craniofacial engagement was evaluated with magnetic resonance imaging (MRI) or CT in 11 patients. The extent of skeletal involvement is described in Table 2. Craniofacial bone involvement was found in 12/16 patients, and almost half of these (5/12; 42%) had compression of optic nerve. Of the five affected, one subject had lost vision unilaterally due to optic nerve damage at 6 years of age, and another two presented with unilateral exophtalmos at the ages of 4 and 9 years. The most commonly affected craniofacial bones were the maxillar (*n* = 9) and orbital (*n* = 8) bones.

**Table 2:** Affected bones, fractures, and surgical operations in pediatric patients with polyostotic fibrous dysplasia (*n* = 16).

Patient number	Age category (years)^a^	Height SDS^a^	Affected bones	Craniofacial involvement	Fractures	Orthopedic (or other) surgery	Orthopedic disabilities/problems	Chronic pain/head aches
1	10–15	−0.5	Ribs, ilium, and femur	−	+	Fixation of femoral fracture	−	

2	15–20	+0.2	Orbital and parietal bones	+	−	−	−	

3	15–20	−0.5	Pelvis and femur	−	+	Epiphysiodesis and spondylodesis	Disabling deformity of pelvis and hip	

4	10–15	−0.2	Orbital, maxillar, frontal, sphenoidal, temporal, mandibular bones, humerus, fibula, tibia, and femur	+ Stenosis of nervus opticus	+	Fixation of femoral fracture; osteotomy	−	+ Headaches

5	15–20	+0.4	Sphenoidal, frontal and orbital bones unilaterally	+ Stenosis of nervus opticus	−	(Decompression of optic nerve)	−	+ Headaches

6	15–20	−1.7	Skull base, parietal, occipital, orbital, temporal, humerus, fibula, tibia, and femur; all unilaterally	+	+	Fixation of femoral fracture times 3; correction of coxa valgus deformity	−	+ Headaches

7	15–20	+0.5	Occipital, parietal, maxillar, mandibular bones, humerus, radius, femur, and tibia	+	+	−	−	

8	5–10	N/A^b^	Ceiling of orbita, maxillar, mandibular, frontal, ethmoidal, sphenoidal, temporal, occipital, and parietal bones, femur, tibia, and fibula	+	+	Fixation of femoral neck bilaterally; osteotomy	Severe scoliosis	

9	15–20	+0.1	Sphenoidal, maxillar, and ceiling of orbita unilaterally; frontal bone, foramina optica bilaterally	+ Stenosis of nervus opticus	−	−	−	+ Headaches

10	15–20	−1.3	Skull base (mostly unilaterally): frontal, sphenoidal, orbital, maxillar, and temporal bones; pelvis, femur bilaterally	+ Stenosis of nervus opticus	+	Femoral osteosynthesis bilaterally	−	Chronic pain

11	10–15	+1.6	Femur and fibula bilaterally; tibia, skull base (widely), mandibular, zygomatic, maxillar bones, humerus, and orbital bones	+ Stenosis of nervus opticus	+	−	Varus malalignment of hip	Chronic pain

12	10–15	−0.3	Frontal, parietal, and ethmoidal bones, concha superior, sinus maxillaris unilaterally, and sinus ethmoidalis bilaterally	+	+	(Skull operation)	−	

13	10–15	+0.5	Maxillar and sphenoidal bones, fossa cranii media	+	−	−	−	

14	10–15	−1.3	Tibia (widely), fibula unilaterally	−	+	−	−	

15	5–10	+1.2	Pelvis, femur, tibia, talus, humerus, and radius	−	+	+ Fixation of femur fracture with osteosynthesis; osteotomy	−	

16	10–15	−0.1	Maxillar bone bilaterally	+	−	−	−	+ Headaches

*SDS, standard deviation score*.

*^a^Age and height SDS at last follow-up visit*.

*^b^Height measure not possible; arm span used as a proxy for height*.

Most commonly affected bones in extra-cranial skeleton were the femur (*n* = 9) and tibia (*n* = 6). Three of the 16 subjects (19%) presented with scoliosis. It was associated with extensive FD lesions at the lumbar spine, pelvis, and hip in one patient, and by FD lesions at ileum in another patient. The third affected patient had pronounced neuromuscular scoliosis. Altogether 11 (69%) patients (7/8 of those with MAS) had sustained at least one fracture by the end of follow-up; most of them had had several fractures in the same affected bone. All but one of those without fracture history had FD lesions only in multiple bones at cranial or facial area. In turn, only one of the five patients with solely craniofacial involvement had sustained a (traumatic) fracture (in nasal bone). Of those with a fracture history, 8/11 had hypophosphatemia as compared with 2/5 of those without a fracture. In six patients (55% of those with a fracture), the fracture(s) were surgically treated. In total, nine patients (56% of all) had needed at least one orthopedic surgery because of the FD lesions (Table 2).

Recurrent headaches were reported by 5 of the 12 patients with craniofacial FD lesions. Recurrent pain was reported by altogether seven (44%) patients. The most severe skeletal manifestations of polyostotic FD were recurrent fractures in the weight-bearing long bones, mostly in the proximal femur.

### Endocrine Features and Growth

Altogether 8 (5/8 girls, 3/8 boys) of the 16 patients with polyostotic FD (50%) were defined as having MAS (Table 1). Among those with MAS, five girls presented with PP and three boys with abnormal testicles, characterized as having firm, irregular consistence, and abnormal structure in testicles either unilaterally or bilaterally. In two of these, testicular ultrasound showed microcalcification. These testicular changes were not associated with abnormal testosterone production and timing of pubertal development was normal in these patients.

**Figure 1:**
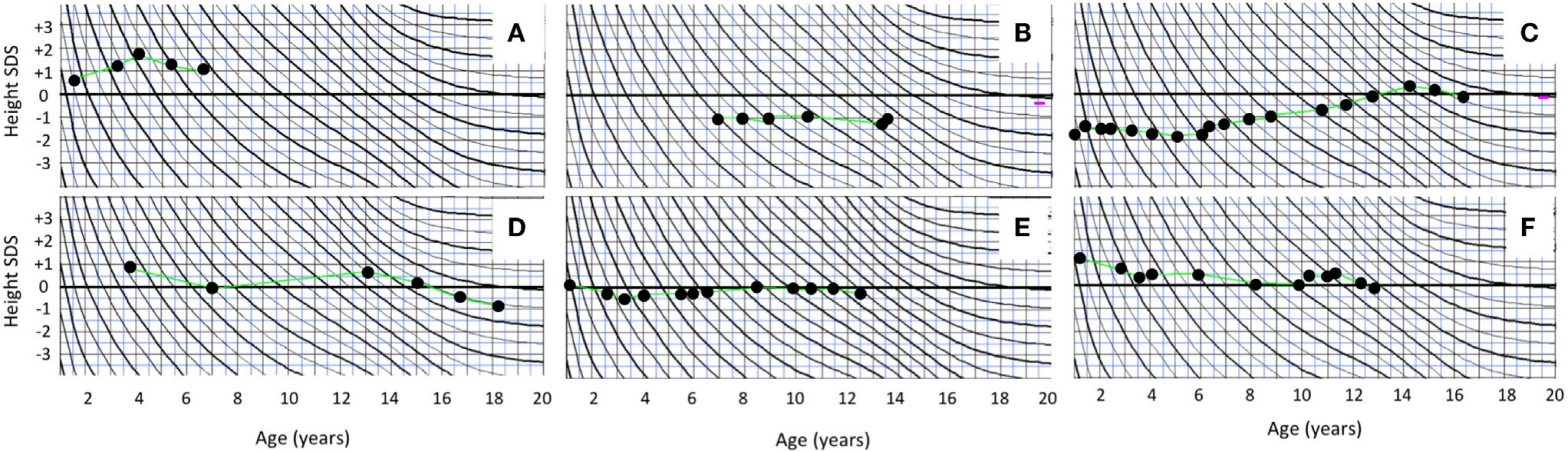
Growth curves of the boys with polyostotic fibrous dysplasia with MAS **(A)** and without MAS **(B–F)**.

**Figure 2:**
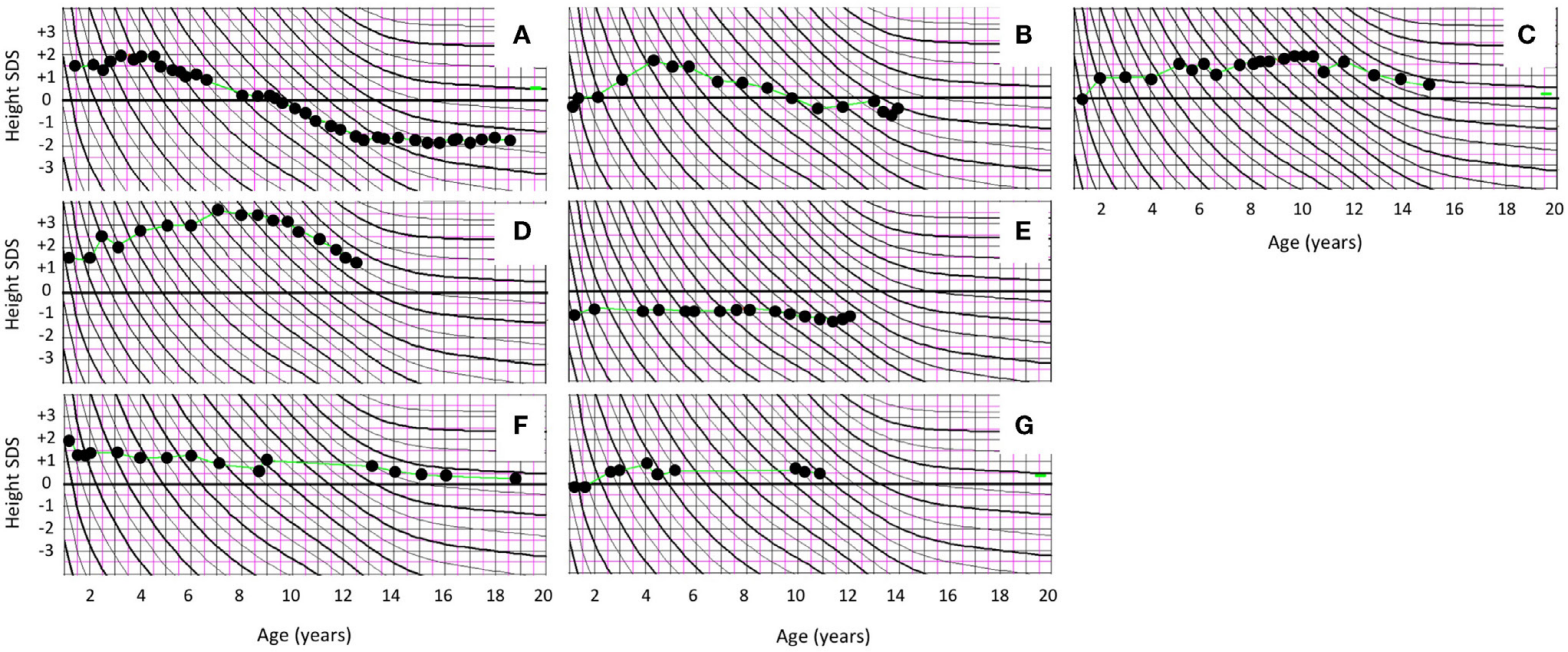
Prepubertal and pubertal growth in height in female patients with polyostotic fibrous dysplasia (FD). Growth curves of the five girls with FD and McCune–Albright syndrome (MAS) manifesting with peripherally driven precocious puberty (PP) **(A–E)**. One of the MAS girls **(A)** had enhanced early growth together with bone age advancement, and she ended up with slightly compromized adult height. Other MAS patients with PP had normal growth and height at the end of follow-up. **(F,G)** Growth curves of the two girls with polyostotic FD but no MAS.

Thyroid function was normal in all subjects; thyroid imaging was not routinely performed in our patients. None of the subjects presented with overt GH excess; circulating IGF-1 levels were within the normal range for age in those with measurements. Growth in height and head circumference were closely monitored, and none had a growth curve suggestive of GH excess (Figures 1 and 2).

All subjects had been born full term and with appropriate size for gestational age with median birth length (*n* = 15) of 51 cm (range 48–54) and weight 3.5 kg (3.2–4.6). The median height *Z*-score at FD diagnosis (*n* = 15) was +0.50 (range −1.70 to +3.50) and at the end of follow-up, +0.05 (range −1.8 to +1.6); none of the subjects presented with short stature (height *Z*-score < −2.0). Among the five girls with PP, height *Z*-score was on average +1.5 at FD diagnosis (range −1.0 to +3.5) and −0.20 at the end of study follow-up (range −1.8 to +1.6) (Table 3). Prepubertal and pubertal growth were within the normal reference range in all males (Figure 1), and in female patients without MAS (Figure 2). Two male MAS patients in whom we did not have growth curves available also had normal growth in height: one at −1.5 SD and the other at +2.0 SD curve. One girl with MAS and PP presented with slightly compromised final height, but still within the normal range, while other girls had normal pubertal growth pattern (Figure 2).

**Table 3:** Height and bone age at commencement of peripheral PP, and at the end of follow-up (at 12–15 years of age), in five girls with McCune–Albright syndrome and PP.

Patient number	Age category (years) at PP	BA advancement (years)^a^ at PP	Height SDS at PP	Treatment of PP	BA advancement^a^ at follow-up (years)	Current height SDS	Parental height SDSs
4	1–4	+0	+0.8 (+1.6 at dg)	Clomifene (4 years–>); leuprorelin (7 years–>)	−1.4	−0.5	Fa −0.3 Mo +0.6

6	1–4	+1.2	+2.0	Clomifene (3 years–>); leuprorelin (11 years–>)	−2.2	−1.8	Fa +1.0 Mo +0.0

7	5–8	+0	+1.4	No treatment	+0.2	+0.5	Fa −0.3 Mo +0.8

11	<1	N/A	N/A (+3.5 at dg)	No treatment	+2.2	+1.6	Fa −0.3 Mo −0.1

14	1–4^b^	N/A	−0.9	No treatment	N/A	−1.1	Fa −0.3 Mo +0.1

*^a^BoneExpert according to G-P; BA advancement = BA − CA*.

*^b^Tanner B2 by 4 years of age, but spontaneous recovery to B1 before progressive pubertal development*.

*PP, precocious puberty; BA, bone age; CA, calendar age; Fa, father; Mo, mother; N/A, not available*.

Pubertal timing was within the normal range in all males and all females without MAS. The three girls with polyostotic FD but no MAS had the menarche at 12.5, 12, and 13 years.

Two of the five PP girls were treated with a combination of estrogen blocker (clomifen) and GnRH agonist (leuprorelin). The remaining three MAS girls with PP were followed without hormonal treatment for PP. In one of these, fluctuation of circulating estrogen level could be demonstrated biochemically. In the two girls treated with clomifen and leuprorelin, vaginal bleeding could be prevented, and further bone age advancement could be blocked. Both of them also reached normal post-pubertal height (Figure 2). PP girls without hormonal treatment also had heights within the normal range at pubertal or post-pubertal age (Figure 2). The treatment and growth characteristics of the PP girls are shown in Table 3.

Brain MRI or CT was performed as part of diagnostic work-up or in the follow-up of 11 of 12 subjects with craniofacial lesions. None had pituitary adenomas or other neoplasms on imaging.

### Other Clinical and Biochemical Features

All eight subjects with MAS presented with café-au-lait spots. Altogether 11 of our 16 patients had presented with mild hypophosphatemia (plasma phosphate below the reference range for age) and hyperphosphaturia. Serum FGF-23 was measured in four of seven Finnish patients, and it exceeded the upper reference range for age in all but one of them. None of the patients had received treatment for mild hypophosphatemia as none presented with muscle weakness or other symptoms of hypophosphatemia, or radiographic features of hypophosphatemic rickets.

One female patient had congenital hepatopathy with neonatal, transient hepatomegaly, and elevated liver enzymes, and she later developed inflammatory bowel disease at adolescence. No other subjects had a history of any hepatic, biliary, or pancreatic disorders or biochemical abnormalities in liver function. Abdominal ultrasound or MRI was performed in additional three of the seven Finnish patients for other reasons, for example, due to unspecific abdominal pain, and they all showed normal findings. Abdominal ultrasound or other imaging studies were not routinely performed in our cohort patients.

Three patients required psychological assessment and support. All these patients reported also recurrent headache or other pain.

## Discussion

This study reports the clinical spectrum of polyostotic FD in a non-selected pediatric cohort. Our cohort included all pediatric patients with polyostotic FD in two Nordic tertiary referral centers between 1996 and 2017.

As expected, the spectrum of skeletal manifestations was wide, from few lesions with mild or no symptoms to wide-spread disabling disease with pain and joint involvement.

Polyostotic FD was part of MAS in half (8/16) of the patients. All of them presented with café-au-lait spots. In addition, approximately two-thirds of girls had PP, and one-third of boys had testicular involvement, with no other endocrinopathies. None of the patients manifested with hyperthyroidism or overt GH excess. Mild hypophosphatemia as a result of FGF-23-mediated hyperphosphaturia was evident in two-thirds of the patients. In the largest reported FD cohort (NIH cohort) by Collins and coworkers, PP was found in 50% of the female patients and gonadal abnormalities in 70% of the male patients. In the same cohort, café-au-lait spots were found in 66%, renal phosphate wasting in 43%, thyroid abnormalities in 66% (28% with hyperthyroidism), and GH excess in 21% of the patients (8). The prevalence of skin manifestation and PP in our cohort is well in agreement with that study, while the lower prevalence of testicle and thyroid abnormalities might be explained by the lack of routine ultrasound of testicles and thyroid gland in our cohort. However, there was a clear difference in the prevalence of hyperthyroidism and GH excess, which were found in one-fourth of the NIH cohort patients but in none in our cohort. The lack of a wider spectrum of endocrinopathies in our cohort is probably partly explained by small sample size, and partly by the non-selected population including also patients with milder form of the disease. Our cohort is likely to be a more representative sample of pediatric patients with polyostotic FD in tertiary hospitals while the NIH cohort may be biased toward more complicated patients.

Our finding of macro-orchism and testicular microcalcification without abnormal hormone secretion has been reported in a few previous studies (13, 14). Serum IGF-I and growth pattern in height and head circumference were closely followed in our pediatric patients, but none of them had signs of GH overexpression. Lack of GH excess in our sample may be partly explained by the young age of our patients. In a previous study, the median age at the diagnosis/manifestation of acromegaly was 24 years of age, and GH excess was associated with accelerated growth in 85% of pediatric patients (9). Moreover, acromegaly was almost always associated with skull base FD, and half of the patients had adenoma in CT or MRI. By contrast, none of our patients with craniofacial FD had pituitary abnormalities in cranial imaging studies. However, the possibility of GH excess warrants close monitoring of the patients with cranial base involvement (9).

The presenting complaint in our patients with polyostotic FD was variable. Café-au-lait skin spots had assumingly been the first manifestation in most MAS patients, but they often remained unnoticed by both parents and doctors. In girls with MAS, PP was usually the first overt manifestation of the disease. In one female patient, the diagnosis of FD was reached several years after PP, since the girl had no clinical signs of skeletal manifestation at the time of PP, and skeletal imaging/scintigram was not performed upon onset of PP. On the other end of disease spectrum, the presenting complaints in polyostotic FD patients without MAS included fractures, abnormal gait, impaired vision, and headache. Due to the wide spectrum of possible first symptoms, polyostotic FD with or without MAS still remains a diagnostic challenge. It is notable that café-au-lait spots could have revealed all the MAS patients already at young age in this cohort. Thus, the diagnosis of MAS should be considered in patients with wide-spread café-au-lait changes, especially if typical for MAS (“coast of Maine”), even without any other clinical signs of the disease as these may develop much later. MAS should also be kept in mind as an alternative diagnosis in patients evaluated for neurofibromatosis. As for those with polyostotic FD without MAS, craniofacial asymmetry and plain radiographs in case of fractures could usually lead to the diagnosis.

Majority of the patients in our cohort had sustained a fracture (11/16). This is in agreement with a previous study on fracture risk in FD (15). As assumed, several refractures often occurred in the bones with fibrotic lesions. Especially femoral fractures often needed one or multiple surgical fixations. Hyperphosphaturia was slightly more common in those with a fracture history than in those without fractures, but no definitive conclusions can be drawn because of the small sample size, especially since some patients had only craniofacial lesion with low fracture risk. One of the five patients with optic nerve compression had lost vision unilaterally, which underlines the need for MRI follow-up in craniofacial FD. Other symptoms affecting the quality of life were recurrent headaches in almost one-third of our patients. The relatively high rate of mental problems related to chronic pain in our cohort is in line with a recent questionnaire study in a large Dutch FD cohort showing impaired quality of life compared with general population (16). Pain is a common feature in FD and may be underestimated especially in pediatric patients (17).

To the best of our knowledge, this is the first study to report growth characteristics in pediatric patients with polyostotic FD or MAS. In our cohort, all subjects were born with weight and height appropriate for gestational age. On average, height *Z*-scores were within normal reference range at diagnosis and at the last follow-up visit. Prepubertal and pubertal growth pattern was normal in all subjects without MAS, suggesting that their gonads are not affected by the activating *GNAS* mutation. On the other hand, over half of our MAS patients with peripherally driven PP were followed without treatment, and they also had normal growth and seemed to reach normal adult height. In the two PP girls with more severe PP, the outcome of treatment with estrogen blocker and GnRH agonist was satisfactory, although one of the girls ended up with slightly shorter final height than expected by parental height *Z*-scores.

The best treatment modality for peripheral, gonadotropin-independent PP in MAS patients has remained unclear (6, 18). A recent and largest study on PP treatment in MAS reported safe and beneficial effect of aromatase inhibitor letrozole on skeletal maturation, growth velocity, and predicted adult height (19). In our retrospective cohort, two girls with PP had been treated with a combination of antiestrogen and GnRH agonist; they were both treated before the era of aromatase inhibitors in the PP treatment. Most of the PP girls in our series did not need any hormonal treatment for PP due to slow progress of puberty. This indicates that the early peripherally driven puberty is not always rapidly progressive in MAS, and watchful wait may be the best option for them. On the other hand, if untreated estrogen excess leads to early maturation of the central pubertal axis, treatment may be challenging with the combination of antiestrogen and GnRH agonist.

In general, the prevalence of MAS is estimated to be 1 per 100,000–1,000,000 (6). With the background population of our pediatric/young adult cohort being approximately three million people, our cohort of eight MAS patients gives an estimated prevalence of 1:375,000, which is within the previously reported range. This indicates that our cohort represents the vast majority of pediatric MAS patients in these two countries. As for polyostotic FD, our cohort includes all severely affected patients, but some milder cases may be missed, if they have been treated and followed in smaller institutes. Although our cohort included the majority of MAS and polyostotic patients in our population, the sample size is small, which is the main weakness of the study.

In conclusion, MAS still remains a diagnostic challenge, and delayed diagnosis may have significant consequences. The prevalence of thyroid and GH excess seems to be low in pediatric MAS patients but follow-up until adulthood with careful endocrine assessments is warranted. Skeletal outcome of polyostotic FD can be severe and disabling, and craniofacial lesions can even lead to permanent vision loss. Therefore, craniofacial FD should be closely monitored with skull MRI in childhood to enable early detection of optic nerve compression. Chronic pain is a significant complication and needs to be assessed during follow-up visits in children with polyostotic FD. Pediatric patients with polyostotic FD and particularly with MAS should be followed in tertiary hospitals with experience on these rare patients with strikingly broad spectrum of disease manifestations.

## Ethics Statement

This study was carried out in accordance with the Declaration of Helsinki. This retrospective registry-based study protocol was approved by the Research Ethics Committees of Helsinki University Hospital and Karolinska University Hospital, and patient consents were obtained as necessitated by these permissions.

## Author Contributions

PU collected and analyzed the data and wrote the manuscript text with the help of other authors; HV provided the Finnish patients, collected patient data, and participated in writing the manuscript; SB provided the Swedish patients and participated in writing the manuscript; OM planned the study and the manuscript structure and participated in writing the manuscript; and EH provided the Swedish patients, collected patient data, and participated in writing the manuscript.

## Conflict of Interest Statement

The authors declare that the research was conducted in the absence of any commercial or financial relationships that could be construed as a potential conflict of interest.
